# Risk Assessment of China Rapeseed Supply Chain and Policy Suggestions

**DOI:** 10.3390/ijerph20010465

**Published:** 2022-12-27

**Authors:** Fujia Li, Kexin Guo, Xiaoyong Liao

**Affiliations:** 1 Institute of Geographic Sciences and Natural Resources Research, Chinese Academy of Sciences, Beijing 100101, China; 2 College of Resources and Environment, University of Chinese Academy of Sciences, Beijing 100049, China

**Keywords:** rapeseed production, supply chain, security risk, policy suggestions

## Abstract

Rapeseed, as the most important oil crop in the world, not only affects national food security but also affects energy security and environmental security. It is very important to conduct a risk assessment of China’s rapeseed supply chain and put forward suggestions to construct a safe, effective, and accessible supply chain. In order to accurately evaluate the safety of the rapeseed supply chain from 2010 to 2020, we applied fuzzy multiconnection theory and analytic hierarchy process model (AHP). A comprehensive risk assessment model for the rapeseed supply chain with two primary indicators and 10 secondary indicators was constructed. By establishing the rapeseed risk evaluation model, we quantitatively analyzed the risk of China’s rapeseed supply chain. The domestic risk of production is still high, and the international risk under the high import dependence is alarming. We put forward risk prevention and countermeasures for China’s rapeseed supply chain. The results show that China has a large demand for rapeseed products, but the increase in China production is limited and the import from other countries is unstable. The proposed suggestions are designed to optimize and enhance the stability of the rapeseed product’s supply chain. It is recommended to continue to consolidate and deepen the cooperation with traditional trading partners such as Germany, Spain, the United States, and Brazil; expand other import sources to build a more diversified and efficient rapeseed product import network and extend the supply chain of rapeseed products. This research can be a basis for making decisions for promoting the sustainable and efficient development of the rapeseed supply chain.

## 1. Introduction

**Figure 1:**
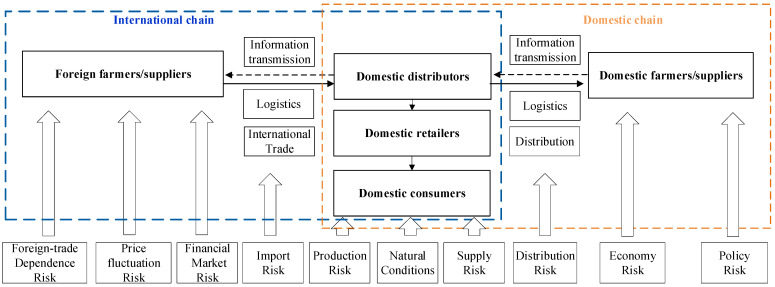
Structure of the rapeseed supply chain.

Rapeseed is the most important oil crop in China [1]. Facing anxious energy and environmental problems, the security of rapeseed supply not only affects food security but also affects energy security and environmental security [2]. Since 2016, the foreign-trade dependence on rapeseed was maintained at 14–20%, showing high self-sufficiency [3]. In fact, in the past decade, the production in China has remained between 13 and 14.5 million tons, without significantly increasing. With the cancellation of the rapeseed purchase policy, the planting area has gradually decreased from 7.49 million hectares in 2000 to 6.57 million hectares in 2019, and the yield of rapeseed cultivation has decreased [4]. If it further decreases, the supply of rapeseed will be negatively affected. Meanwhile, rapeseed importation with high quality and low price has a great impact on China’s rapeseed planting income [5,6,7], resulting in the farmers in China having low planting intention. In the short term, the domestic rapeseed yield cannot be greatly increased, and the current trend of anti-globalization and trade protectionism has led to more uncertainties in global trade [8,9,10,11,12]. This further escalates the uncertainties of China’s rapeseed product import [13] and increases the probability of sudden product shortage, posing great challenges to China’s rapeseed product supply chain (Figure 1). Due to the increasing demand for rapeseed, rapeseed substitutes such as soybean and oil products, whose supply is highly dependent on importation from foreign countries in China, also face increasing international supply pressure [14].

Therefore, the risk of China’s rapeseed supply chain is becoming increasingly concerning. It is urgent to carry out policy at the national level, optimize the structure of China’s rapeseed product supply chain, and improve the ability to cope with the risk of sudden changes in international trades.

Identifying the risk and putting forward countermeasures is critical to keep the rapeseed supply chain stable and establish an efficient rapeseed supply chain [15]. Therefore, we established risk assessment model, and we analyzed the risk of China’s rapeseed supply from 2010 to 2020. Then, we put forward the risk prevention and control countermeasures for China’s rapeseed supply chain.

## 2. Literature Review

There have been many previous studies on risk identification, risk assessment, and risk management in the supply chain. Kraljic earliest identified the risk of the supply chain considering the uncertainty and the supply interruption caused by external factors [16]. Since then, scholars have studied the framework of supply chain risk identification from different perspectives [17,18,19,20]. The chain structure of the supply chain determines that the risk can spread throughout the whole system. The flowchart model and scenario analysis method are used in most studies to build risk identification framework of a multistage supply chain from the whole system [21,22]. In supply chain risk analysis and evaluation, scholars have realized that the supply chain is a complex system. When identifying risk factors, the parts connecting each entity should be included in the whole risk identification system. The analytic hierarchy process (AHP) model, principal component analysis, and data envelopment analysis (DEA) are widely used in risk assessment [17,23]. To quantify the qualitative factors, we combine multiple fuzzy theories with the AHP model [24]. This allowed evaluating the risk of the rapeseed supply chain by integrating deterministic and uncertain factors.

The risk in the supply chain of agricultural products includes endogenous factors and exogenous factors, and the risk at one stage may bring losses to the whole food supply chain [25]. Exogenous factors include natural environment risk, economic environment risk, policy environment risk, and legal environment risk. Davis studied the impact of natural environmental risks on the supply chain [26]. Gao et al. analyzed the impact of economic environmental risks on the supply chain [27]. For example, the infrastructural constraints in agricultural production exacerbate transportation costs and risks, thus resulting in a lower income for agricultural producers. Endogenous factors are risks caused by the inevitable conflicts of interest and information asymmetry among the nodes of the supply chain. Risks are transitive on the food supply chain. However, there are still few studies on specific agricultural supply chain risks, which is not conducive to the risk avoidance of specific products and can easily affect the overall market of agricultural products.

Studies on the rapeseed supply chain in China are scarce. Regarding the research, three streams of the literature are most relevant. One stream analyzes the rapeseed industry development as a function of the production and demand structure [28,29,30]. The second stream researches the spatial structure of rapeseed production [31,32,33,34]. The third stream analyzes the development status and potential of the rapeseed industry [35,36,37]. However, there are few studies that identified and evaluated the risks of the rapeseed supply chain. The risks of the rapeseed supply chain are complex and correlated. Previous studies focused more on a single risk and less on the systematic sorting and accurate identification of domestic and foreign food supply security risks. We divided the rapeseed product risk into domestic risks and foreign risks. Domestic risks mainly included product risks, distribution risks, supply risks, natural conditions risks, and policy risks. International risks mainly included the risk of foreign-trade dependence, the risk of import concentration, the risk of price fluctuation, and the risk of financial market (Table 1).

## 3. Materials and Methods

### 3.1. Data Collection

This research explores the risks of the rapeseed supply chain by analyzing the internal risk and external risks. The production and consumption data are from the database of China’s Development Research Center of the State Council. Price data are from China’s National Bureau of Statistics. The foreign-trade dependence degree is calculated from the data on import and export of rapeseed products in each country from the Food and Agriculture Organization (FAO). The price risk data are obtained from the international prices of rapeseed products released by FAO’s Food Outlook report.

### 3.2. Risk Assessment Model

The supply chain consists of foreign suppliers, domestic suppliers, and distributors during international procurement, transportation, and consumption process [26,38,39]. From the perspective of the domestic supply chain, production and distribution, natural environment, supply, policy, and economy jointly determine the supply risks of the domestic supply chain, which is directly related to the supply and demand gap of rapeseed products. The international supply chain mainly includes foreign-trade dependence risk and import concentration risk [40], as well as systemic risks such as price fluctuation and exchange rate fluctuation [41].

Considering the nondeterministic qualitative factors, the fuzzy comprehensive evaluation method was used to evaluate the risk of rapeseed supply chain (Table 2). We emailed and invited more than 100 experts to score the risks of rapeseed policy and the risks of foreign-trade dependence and import concentration online. We collected first-hand data on the production, natural condition, and distribution of more than 50 rapeseed planting bases in Hunan, Guangdong, Henan, Shaanxi, Hubei, Anhui, etc. More than 200 rapeseed traders completed paper questionnaires to score the risks of supply, economic, market, and price of rapeseed. Then, they scored the risks with the same set of comments V = {v1, v2, v3, v4, v5} = {safer, safe, unsafe, unsafer, unsafest}. The corresponding evaluation vector was D = {d1, d2, d3, d4, d5} = {100, 80, 60, 40, 20}. The analytic hierarchy process (AHP) was used to calculate the index weight W. Firstly, single-factor fuzzy evaluation was carried out. We used the fuzzy statistic method to calculate the fuzzy membership matrix R. The fuzzy membership matrix R was calculated according to the proportion of people scoring each comment in comment set V. The fuzzy comprehensive evaluation vector B was calculated as follows: B = W × R. The multifactor comprehensive score of domestic and international rapeseed supply chain was calculated as follows: E = B × D^T^, where E is the comprehensive score, B is the comprehensive evaluation vector, and D^T^ is the transpose matrix of the evaluation branch vector. According to the principle of maximum membership, the fuzzy comprehensive evaluation results correspond to the comment set D: 100–80 as safer, 80–60 as safe, 60–40 as unsafe, 40–20 as unsafer, and 20–0 as unsafest.

## 4. Risk Assessment of the Rapeseed Supply Chain in China

Using the weight vector W and fuzzy membership matrix R, vector B was calculated. The domestic rapeseed supply chain risk evaluation vector was B_d_ = (0.2972, 0.3338, 0.1967, 0.1611, and 0.1122). The score of the domestic rapeseed supply chain was E_d_ = 74.89. According to the comment set D, the domestic rapeseed supply chain could be considered safe. The international rapeseed supply chain evaluation vector was B_f_ = (0.2239, 0.2818, 0.1847, 0.1522, and 0.15738). The score of the international rapeseed supply chain was E_f_ = 65.25. This shows that the international rapeseed supply chain in China could be considered safe but with a low score.

The domestic supply chain and international supply chain are closely related but cannot be completely dependent on one another. In the domestic supply chain, the production, manufacturing, transportation, and consumption of raw materials are all completed in one country, which can quickly balance supply and demand through inter-regional regulation. Agriculture production is unstable, and the agriculture supply chain is where any risk is equally applicable across a country. This is unlike the situation for the international supply chain that can mitigate the risk of a poor harvest in a country through supplies from the rest of the world. In addition, a country’s food production is continuous, and the harvest is seasonal. Regional differences in harvest between different countries in the world can provide continuous supply to meet the production and consumer demand throughout the whole year (China is a northern hemisphere country, and most of the rapeseed harvest season is from May to August, while that in a southern hemisphere country, e.g., Australia, is from October to November, which can guarantee China’s demand and supply throughout the year). However, the result suggests that the proportion of rapeseed consumption that China imports from other countries has increased. Facing frequent extreme weather and the COVID-19 pandemic, the international supply is alarming. This has also affected the domestic supply chain in China.

Combined with the evaluation matrix and index weight model, we obtained the result of the rapeseed supply chain evaluation, which reflected several stages in the rapeseed supply chain.

### 4.1. China’s Rapeseed Product Self-Sufficiency Is High but the Potential for Improvement Is Low; It Can Only Weakly Cope with the Interruption of International Trade

Rapeseed is the most important oil crop in China, which has multidimensional utilization, e.g., for flowers and as vegetable oil, fertilizer, and feed. As proposed by the Food and Agriculture Organization of the United Nations (FAO), in 2020, China produced 6.357 million tons of rapeseed oil and 9.619 million tons of rapeseed, accounting for 21.9% and 23.4% of the global total, respectively. The consumption of rapeseed oil and rapeseed was 8.25 million tons and 11.51 million tons, accounting for 29.2% and 28.1% of the global total, respectively. China is a major producer of rapeseed oil and dreg; however, it has been producing less than it consumes for more than a decade, relying on stocks and imports to make up the gap.

**Figure 2:**
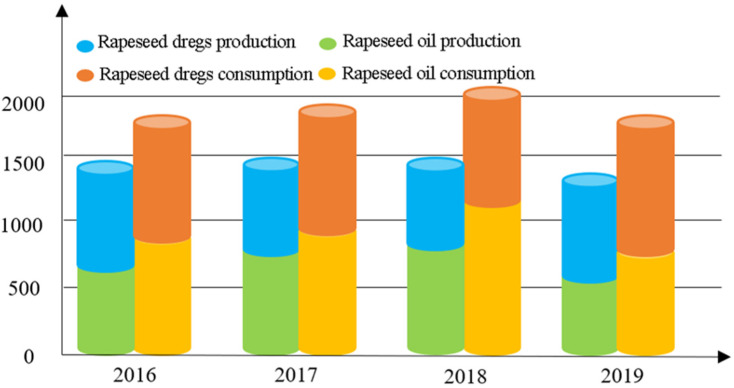
Production and consumption structure of rapeseed products in China (data are from the database of China’s Development Research Center of the State Council).

**Figure 3:**
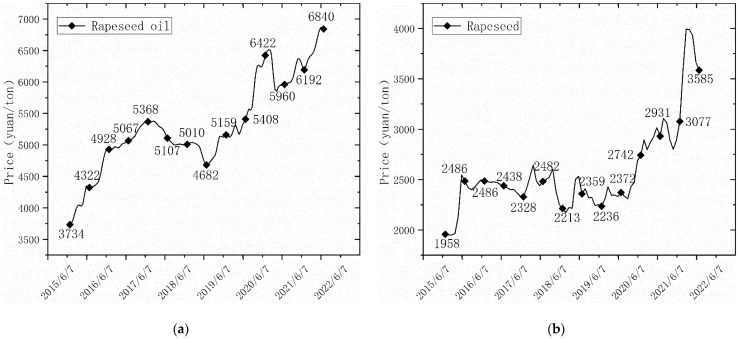
Rapeseed product price trend in China: (**a**) China rapeseed oil price trend: the trend of rapeseed oil price in China, 2015–2022; (**b**) China rapeseed price trend: the trend of rapeseed price in China, 2015–2022 (Producer prices are available in FAOSTAT).

Since 2016, the stocks of China rapeseed oil have continued to decrease, while rapeseed dreg stocks have not changed much (Figure 2). Foreign-trade dependence is generally maintained at 14–20% (calculated on the basis of import data published by FAO), showing high self-sufficiency. In fact, China’s rapeseed production has been maintained between 13 and 14.5 million tons, without increasing significantly in the past decade. With the cancellation of the rapeseed purchase policy, the yield of rapeseed cultivation has decreased, and the planted area has gradually decreased from 7.49 million hectares in 2000 to 6.57 million hectares in 2019. It is worth seeing whether a continued reduction in planted area will further increase the risk. Meanwhile, the cost of non-transgenic rapeseed production in China is much higher than the cost of transgenic rapeseed in Canada, the United States, and Europe. In 2020, the average international rapeseed production price was 430 USD per ton, compared with 797 USD per ton in China (available in FAOSTAT). This is due to the China rapeseed being produced with scattered planting and low efficiency. In China, with low mechanized productivity in most rapeseed-producing areas, field management and harvesting are still performed manually. There is a lack of high-quality rape variety, cultivation techniques, and fertilization habits which match mechanical production. Compared with importing transgenic rapeseed with high quality and low prices from other countries, it is less competitive to cultivate rapeseed in China with large labor input and high risk. In particular, after China canceled the rapeseed purchase policy in 2015, without the support of national policy subsidies, farmers were obviously willing to cultivate more profitable plants than rapeseed. The China rapeseed product price has increased (Figure 3). In fact, even with the support of national policy subsidies, the price is not enough to reverse the price gap with the rapeseed import from other countries with high quality and low prices. As long as there are still countries in the world where the production cost of rape is lower than that of China, companies will choose to import rapeseed from other countries to earn more profit. Thus, although the proportion of imports is not high, it shows strong dependence and a weak ability to cope with the sudden shortage in international trade.

### 4.2. Stable Import Source Is Lacking; Structural Optimization Is Urgently Needed under the Situation of Intensified International Trade Conflicts

**Figure 4:**
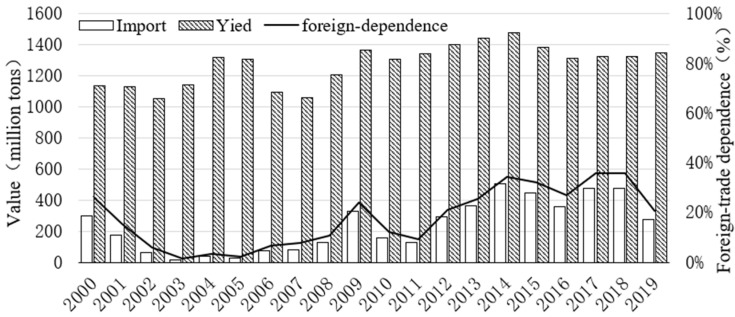
Foreign-trade dependence degree of China’s rapeseed products (calculated using the import data published by FAO).

**Figure 5:**
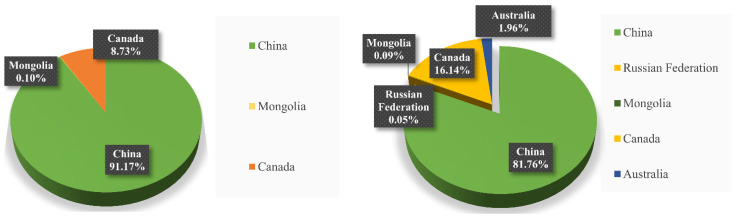
China rapeseed products import countries and proportion, 2010 and 2014 (contains yield in China). Trade data are available in FAOSTAT.

As shown in the trade data published by FAO, since the 21st century, the foreign-trade dependence for China’s rapeseed products decreased from 26.1% in 2000 to 1.5% in 2003. Since then, it has continued to increase, reaching 35.8% in 2018. China’s large demand for rapeseed has maintained its high dependence on international imports, but it can only weak cope with the shortage in international trades (Figure 4). The supply chain structure of Chinese rapeseed products is simple. Rapeseed products are mainly imported from four countries, Canada, Australia, Russia, and Mongolia, with more than 90% from Canada, in contrast to imports from Russia accounting for only 3% of China’s total imports, and imports from Mongolia and Australia accounting for 1% (Figure 5). The lack of import sources increases the fragility of the supply chain of China’s rapeseed products. Accordingly, it cannot cope with sudden changes in the international trade of rapeseed products. In 2019, Chinese customs inspected pests from Canadian rapeseed and canceled the export licenses of three core Canadian rapeseed companies. The import of rapeseed decreased from 4.443 million tons in 2018 to 2.358 million tons, directly causing a shortage of rapeseed in China. By the time rapeseed importation from Canada resumed in 2020, the duty-paid price of rapeseed imports had risen from 3066 RMB per ton, the lowest point on 7 May 2019, to 4724 RMB per ton, the highest point within the year, on 25 December 2020, with a sharp increase of 35.1% (data available in FAOSTAT). This indicates that China’s emergency support capacity for rapeseed products is seriously insufficient when facing the sudden situation in international trade.

With the extent of anti-globalization and trade protectionism, the risk of China’s rapeseed product import has further escalated. The probability of foreign-trade interruption has increased. It This a great challenge to China’s rapeseed product supply chain. Under the increasing pressure of the international supply chain, the demand for alternative rapeseed products, such as soybean, which is highly dependent on foreign countries, is also on the rise. Therefore, it is urgent to carry out policy at the national level, optimize the structure of China’s rapeseed supply chain, and improve the supply ability to cope with the risks of sudden situations in international trade.

### 4.3. Russia–Ukraine Conflict, COVID-19, and the Pressure of Carbon Peaking and Carbon Neutrality Goals Have Combined to Create Severe Challenges for the Global Rapeseed Supply System

The Russia–Ukraine conflict has hindered sowing, destroyed agricultural and other infrastructure, and stopped logistics channels, hampering production, affecting the global rapeseed supply. Ukraine is the major producer and exporter of rapeseed, accounting for 20% of the global total. Dnipropetrovsk and other plains in southern Ukraine are under intense tension between Russia and Ukraine, which are the main rapeseed-producing areas. The conflict has caused destruction of the agricultural facilities and infrastructure, and farmers have stopped sowing, directly affecting the production of rapeseed. Meanwhile, the main export routes of Ukraine, Mariupol, and other Black Sea ports have been blocked, and transportation has been disrupted, hampering the export of rapeseed products. The conflict has already impacted global markets. Reopening and repairing the damaged inland transport infrastructure and seaports, as well as food storage and processing infrastructure, will continue to put pressure on international rapeseed supply markets over the next 2–3 years.

COVID-19 has caused a shortage of labor, capital, and other factors, having a huge impact on the global production and supply of rapeseed; accordingly, global rapeseed production declined for two years. In 2020, rapeseed planting area and production decreased in the European Union, while rapeseed planting area in Canada also decreased to 20.8 million acres, down 0.8% from a year earlier. At the same time, with the aggravation of global climate change and the pressure of reducing carbon emissions, the demand for rapeseed oil for biodiesel production is increasing year by year in major rapeseed product-consuming countries. This necessitates the importation of more global rapeseed products and increases the import cost of rapeseed products in China. The goal of carbon peaking and carbon neutrality will also increase industrial demand for rapeseed oil, which will further increase the pressure on the supply chain of rapeseed products.

We identified and analyzed the risk of the rapeseed supply chain in China. There are many risks involved in China’s rapeseed supply chain, and the inducement and influence of each risk are different. From the perspective of risk management, the measures to deal with risks include risk diversification, risk avoidance, risk prevention, and risk reduction. Different risk management methods should be adopted for different types of risks (Table 3). On the basis of the evaluation results of China’s rapeseed supply chain, we put forward optimization directions and policy suggestions for the Chinese rapeseed supply chain.

## 5. Discussion

### 5.1. The Optimization Direction of the Rapeseed Supply Chain in China

#### 5.1.1. The Demand for Rapeseed Products in Major Global Consumer Countries Is Significantly Different, Which Affects the International Trade Pattern and Provides Potential Space for China to Optimize Import and Export Channels and Improve Supply Chain Security

Rapeseed in China is mainly used for human consumption and, thus, has high requirements for quality. Currently, China mainly imports rapeseed products from Canada, Russia, Australia, and Mongolia, with Canada accounting for up to 86.13%. In addition, China is the second largest importer of rapeseed oil and rapeseed dreg, with 59.64% of rapeseed oil and 90.41% of rapeseed dreg coming from Canada (data from FAO). The high dependency on importation from Canada poses a great risk. The European Union (EU) has a relatively low demand for rapeseed oil and rapeseed dreg; thus, the import volume is small. Rapeseed is mainly imported to produce biodiesel, thus having low requirements for quality. EU is the largest rapeseed-importing group in the world, mainly importing rapeseed from Central and Eastern Europe and Central Asia, which are important elements of the land Silk Road in China’s “One Belt, One Road” strategy. This region has a large area of arable land, but the reclamation is insufficient, and there is a lack of efficient agricultural technology. It has great potential for large-scale cultivation and production of high-quality rapeseed products in the future. At present, the region mainly supplies the European Union with low-quality rapeseed to make biodiesel, with limited profits. Due to the big difference in the import demand for rapeseed products between China and the EU, China should optimize the structure of rapeseed product importation and adopt effective measures to carry out agricultural cooperation with the above regions, providing them with technical support, capital, and equipment, while focusing on the import of manufactured products such as rapeseed oil and rapeseed dreg from these regions. This will greatly stimulate the local initiative to plant high-quality rapeseed products and increase agricultural profits, thus achieving the strategic goal of win–win cooperation under the Belt and Road Initiative, as well as opening up stable supply sources of large-scale rapeseed products for China.

#### 5.1.2. The Belt and Road Productivity Cooperation Will Provide Strong Support for China to Open up New Import Sources for Rapeseed Products

Countries and regions in Central and Eastern Europe and Central and Western Asia along the Belt and Road, such as Belarus, Kazakhstan, Ukraine, Russia, and Arabia, are the main rapeseed sources in Europe, while European countries have a relatively low demand for rapeseed oil and rapeseed dreg from this region. Moreover, there is no habit of rapeseed oil consumption in these regions; thus, domestic rapeseed demand is limited. The demand for rape products is different in China; accordingly, there is huge supply potential for China. Russia, Kazakhstan, Saudi Arabia, and other Central and West Asian countries have a large amount of idle arable land, which has large potential to increase rapeseed yield but a shortage of investment funds. Furthermore, they have a strong willingness to cooperate in cross-border agricultural productivity, technology, and finance. In addition, they have the advantages of large capacity, long distance, and low cost for importing from countries along the belt and road, with more goods being exported than imported, resulting in an empty train upon return. If China imports rapeseed from these countries, it can make full use of the return trains. Although the conflict between Russia and Ukraine will hinder the transportation and logistics in Ukraine and the surrounding regions in the short term, in the long term, the production potential of rapeseed in Central and Eastern Europe will still provide a good strategic supply for China’s rapeseed. Therefore, taking full use of the “Belt and Road” productivity cooperation and transportation advantages, actively opening up and seeking new supply sources of rape products can provide great potential for diversifying and improving the security of China’s rapeseed product supply.

#### 5.1.3. The Huge Amount of Waste Oil (Gutter Oil) Produced Provides Broad Prospects for China to Develop the Biodiesel Industry and Regulate the Consumption Structure of Rapeseed Products

In recent years, European and American countries have imported rapeseed oil in large quantities and promoted the development of the biodiesel industry through legislation, planning, subsidies, and other policies, resulting in a continuous increase in global rapeseed oil consumption demand. To prevent the impact of the development of biodiesel on the supply chain of rapeseed oil products, the Chinese government restricted the direct production of biodiesel from rapeseed oil and limited the development of the biodiesel industry, adhering to the principle of not competing with others for grain or land. The production of waste oil (gutter oil) in China is huge (about five million tons per year), which provides a possibility for biodiesel development. The biodiesel production from waste oil will not affect the supply chain of rapeseed products, but can greatly reduce the nonedible consumption of rapeseed products, which is conducive to improving the stability of the rapeseed supply chain.

### 5.2. Policy Suggestions to Improve the Stability of Rapeseed Supply Chain

#### 5.2.1. Carry out Cooperation on Rapeseed Cultivation and Agriculture along the Belt and Road, and Expand Supplementary Channels for Rapeseed Product Supply from Russia and Central and Western Asia

It is recommended to strengthen financial cooperation on rapeseed cultivation along the Belt and Road, and expand imports of rapeseed products from these regions through pre-orders and targeted loans for rapeseed products. It is also recommended to encourage large and medium-sized state-owned agricultural enterprises to engage in diversified forms of agricultural cooperation such as contracting land to investment by multinational enterprises. Cooperation with these countries in targeted oilseed rapeseed cultivation will be ideal to help expand the cultivated area in this region and export rapeseed to China. Moreover, it is recommended to adopt a capacity replacement mode, export applicable technical capacity and infrastructure construction to Central Asia and West Asia, replace the equity of local rapeseed products and planting and processing enterprises, and increase the import share. Within 5 years, this will significantly increase the yield and supply of rapeseed products to China from Central and Eastern Europe, Central Asia, and other regions along the Belt and Road, as well as establish a Eurasian support base for China’s overseas supply chain of rapeseed products.

#### 5.2.2. Make Full Use of the Return Trains to Explore the Supply Potential of Rapeseed Products along the Central and Eastern European (CEE) Routes

Using the return trains, it is recommended to provide appropriate tax breaks and preferential mechanisms for rapeseed products exported to China from CEE Belt and Road regions, thereby reducing transportation costs and increasing the share of rapeseed dreg and oil imports to CEE countries. It is also recommended to increase imports of rapeseed oil and rapeseed dreg from CEE countries, relying on large state-owned oil and oil enterprises. On the premise of strengthening quality supervision, China can moderately lift import access to rapeseed from Central and Eastern Europe.

#### 5.2.3. Extend the Supply Chain of Rapeseed Products, Support the Utilization of Waste Oil as Resources to Produce Biodiesel, Restrict the Export of Biodiesel to Foreign Countries, Effectively Reduce the Nonedible Consumption of Rapeseed Products, and Improve China’s Rapeseed Industry Chain with Health and Green Development

It is recommended to establish standardized and strict procedures for waste oil recovery, as well as strengthen the quality supervision of biodiesel produced from waste oil. It is also recommended to establish a national-level biodiesel company, unify the pricing method of diesel purchase and sale, and widely distribute biodiesel gas stations and refueling machines throughout the country. Petrol stations can be supported in promoting biodiesel in the form of targeted support and subsidies. By referring to the European and American models, the compulsory addition of biodiesel can be gradually promoted on a pilot basis, and the proportion of adding biodiesel can be steadily controlled and increased to eliminate the damage to ecology and health caused by China’s gutter oil. Moreover, it is recommended to effectively prevent the flow of gutter oil to the table, greatly reduce the industrial application of rapeseed products, and improve the supply ability of China’s rapeseed products.

#### 5.2.4. Established a Diversified Fiscal and Tax Mechanism and Steadily Expand the Development of the Rapeseed Planting Industry in Winter Idle Fields in the Yangtze River Basin

It is recommended to set up a special state fund to ensure the supply of rapeseed, providing targeted support for the planting of rapeseed in idle fields in the winter in the Yangtze River Basin, and giving incentives to the planting of rapeseed in idle fields in the winter. Due to the lack of high-quality rapeseed varieties and mechanized planting techniques in China, the producer price of rapeseed remains much higher than the price in other countries. To reduce the cost of rapeseed chain, more policies should be implemented, such as providing low-erucic-acid and low-glucosinolate seeds for large-scale mechanized and efficient planting. It is also recommended to support mechanized operations and large-scale planting by giving subsidies. The awards and subsidies for major counties producing rapeseed can be increased, along with investment in scientific research for the development of the rapeseed industry, thus supporting research on high-quality rapeseed scientific and technological breeding, industrial layout, and land-use optimization. Furthermore, it is recommended to support funding and encourage policies for idle field transfer in winter, as well as improve the scope and intensity of policy insurance for rapeseed. Through a series of fiscal and tax policies, the planting intention of farmers can be increased, and the planting yield of rapeseed in idle flown in winter can be effectively increased.

## 6. Conclusions

A combination of qualitative and quantitative analysis was proposed for the assessment of the risks of the rapeseed supply chain in China. Firstly, we constructed a model for the evaluation of the risks of the rapeseed supply chain, establishing two primary indicators and 10 secondary indicators. Due to the uncertainty and coupling effects among various risks, we used the fuzzy comprehensive evaluation to identify the risk of China’s rapeseed supply chain. The domestic rapeseed supply chain could be considered relatively safe, whereas the international rapeseed supply chain in China is alarming. Secondly, on the basis of the evaluation results of China’s rapeseed supply chain, we put forward optimization directions and policy suggestions for China’s rapeseed supply chain.

However, it is important to emphasize that the supply chain is a complex and dynamic mechanism. To better manage the supply chain, the correlation among risks should be further assessed in future research.

## Figures and Tables

**Table 1:** Risk identification of rapeseed supply chain.

Risk Type	Risk	Risk Source
Domestic risk	Production	Risks in farming and primary processing of agricultural products, including product quality and safety risks, production technology risks, and disease risks.
	Distribution	Risks in the agricultural products marketing process, including demand fluctuation risk, product price risk, and distribution channel risk.
	Supply	Risks caused by supply chain interruption in the process of purchasing agricultural supplies by farmers, mainly including the quality of agricultural supplies that directly affects the planting and breeding of agricultural products, and the delay in delivery that leads to missing the best planting and breeding opportunities, resulting in a supply shortage.
	Policy	National policies and laws have an impact on the production and supply of agricultural products. The adjustment of agricultural tax administration, agricultural land policy, and other supportive policies will increase uncertainty in the supply chain.
	Economy	A complex and changeable macroeconomic environment leads to a loss of supply chain deviation from the expected. Inflation not only leads to a decline in purchasing power, affecting the sales of agricultural products, but also raises the cost of agricultural enterprises. The economic cycle also has an impact on the supply chain, and the current economic downturn causes instability in the supply chain.
	Natural conditions	Natural conditions are mainly unpredictable and irresistible risks caused by sudden changes in the natural environment, such as flood disasters, typhoons, rainstorms, pests, and diseases.
International risk	Foreign-trade dependence	Foreign-trade dependence risk refers to the supply uncertainty caused by the high dependence of national commodity demand on foreign supply.
	Import concentration	Concentration risk refers to the high dependence of commodity import in a country or a region.
	Price fluctuation	The large-scale price fluctuation in the international food market not only affects the producer enthusiasm in exporting countries, but also affects the cost of international food purchase in importing countries.
	Financial markets	Financial markets affect supply chains as a function of price mechanisms, market expectations, and food trade.

**Table 2:** The index and weight in the risk assessment model of the rapeseed supply chain.

Target	The First-Level Index	The Second-Level Index	Weight	Fuzzy Membership Degree				
				Safer	Safe	Unsafe	Unsafer	Unsafest
The risk of rapeseed supply chain	Domestic risk (0.5405)	Production	0.2769	0.3	0.35	0.2	0.15	0
		Policy	0.1067	0.4	0.35	0.15	0.1	0
		Economy	0.1241	0.45	0.15	0.35	0.05	0
		Natural conditions	0.1602	0.2	0.3	0.2	0.25	0.05
		Supply	0.1607	0.2	0.4	0.15	0.23	0.02
		distribution	0.1715	0.3	0.4	0.15	0.15	0
	International risk (0.4595)	Foreign-trade dependence	0.3644	0.3	0.25	0.15	0.15	0.15
		Import concentration	0.4137	0.1	0.3	0.25	0.15	0.2
		Price fluctuation	0.1332	0.35	0.2	0.1	0.2	0.15
		Financial markets	0.0887	0.3	0.45	0.15	0.1	0

Weights range from 0 to 1; the total weight of the first-level indicator is 1.

**Table 3:** Risk management and strategies.

Risk Type		Risk Management	Strategies
Domestic risk	Production	Risk diversification	Import sources diversification
	Policy		
	Economy		
	Supply	Risk aversion	Extent the transport channel
	distribution		
	Natural conditions	Risk prevention	Production and storage
International risk	Foreign-trade dependence		
	Import concentration		
	Price fluctuation	Risk reduction	Monitoring and managing with a financial measure
	Financial markets		

## Data Availability

All data were obtained from https://www.fao.org/faostat/en/ (accessed on 16 November 2021).
